# Supplementary material for: Hormone Receptor Expression and Activity for Different Tumour Locations in Patients with Advanced and Recurrent Endometrial Carcinoma
Source: Cancers (Basel). 2024 May 30;16(11):2084. doi: 10.3390/cancers16112084 (PMC11171125; doi:10.3390/cancers16112084)
Supplement: Supplementary file 1 [file cancers-16-02084-s001.zip › cancers-2989483-supplementary.pdf]

Supplementary Materials:

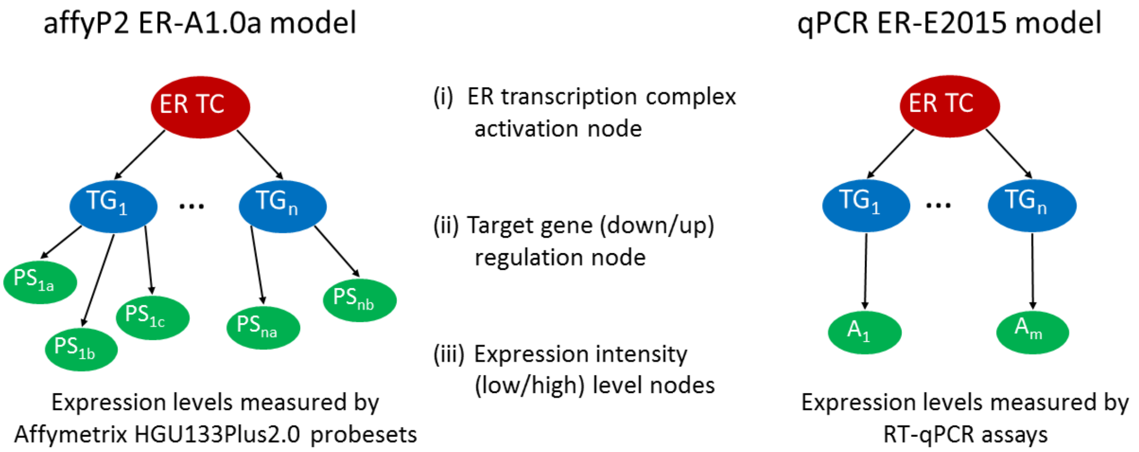

Supplementary Figure S1: Schematic representation of ER pathway model network. Left: Schematic of Affymetrix HGU133Plus2.0 (AffyP2) model network. Centre: Node type description. Right: Scematic of PCR model, RT-qPCR assays were developed for a subset of target genes of the AffyP2 model [24].

Supplementary text S1

Complete protocol for IHC expression procedure and RNA isolation

Complete protocol for IHC expression procedure [24]

ER and PR IHC expression was analyzed on two 4- µm tumor-containing sections of formalin-fixed paraffin-embedded (FFPE) tumor blocks, which were sectioned and mounted on Superfrost slides. After EDTA antigen retrieval and hydrogen peroxide endogenous peroxidase blocking, slides were incubated with either ER*α* antibody (SP1 RM-9101-S, Thermo Scientific Immunologic, Waltman, MA, USA) at 1:40 dilution in normal antibody diluent (Immunologic BV, Duiven, the Netherlands), or PR antibody (PgR636 PR antibody, Dako, Denmark) at 1:500 dilution in normal antibody diluent. The slides were subsequently incubated with PowerVision+ Poly-HRP (Leica Microsystems, Buffalo Grove, IL, US) antibodies at 1:1 dilution in 1x PBS containing 0.05% Tween 20, followed by visualisation with PowerVision DAB substrate solution (Leica Biosystems, Buffalo Grove, IL, US). Finally, the slides were counterstained with hematoxylin. They were dehydrated, and mounted. As an internal control, slides of breast and liver tissue slides with known ER*α* and PR IHC status were stained as described above.

Complete protocol for RNA isolation [24]

In samples from the Radboudumc clinical cohort, marked tissue of interest was microdissected from two consecutive 10-µm FFPE sections. Subsequently, the tissue was transferred into 2-ml microcentrifuge tubes. RNA was extracted using the miRNeasy FFPE Kit (Qiagen, Hilden, Germany) with an optimized protocol. Briefly, the tissue was first incubated for 15 minutes at 60°C and 15 minutes at 80°C with 240 µl buffer PKD and 50 µl Proteinase K, followed by the addition of500 µl buffer RBC. The sample was transferred to a QIAmp mini-spin column (Qiagen, Hilden, Germany) and centrifuged for 30 seconds at 10 000 rpm. The flow-through was mixed with 1200 µl of 100% ethanol, and 700 µl of the sample was transferred to a new RNeasy MinElute spin column and centrifuged for 15 seconds; discarding the flow-through. This step was repeated until the entire sample had passed through, followed by the addition of 500 µl buffer RPE, and centrifugation at 10 000 rpm with a closed lid for 15 seconds. A further 500 µl of buffer RPE was added, and the sample was centrifuged again at 10 000 rpm for 2 minutes. The spin column was then placed in a 2-ml microcentrifuge tube. The sample was centrifuged for 2 minutes with the lid closed and 5 minutes with the lid open, both at 10 000 rpm. The flow-

through was discarded afterwards. The spin column was then placed in a 1.5-ml micro-centrifuge tube, and 30 µl RNase-free water was added directly onto the membrane of the spin column, and centrifuged for 1 minute at full speed with the lid closed. To increase yield and total volume, this step was repeated. The eluate was treated with 7.2 µl DNase solution from the Siemens VERSANT Tissue Prep Reagent kit for 30 minutes at 37°C to ensure complete removal of DNA. .

Supplementary Table S1: Tumour grade, immunohistochemistry analysis, ER-pathway activity score and tumour locations.

Supplementary Table S1. A. ER-IHC in different tumour locations stratified by tumour grade. Predetermined cut-offs of ER-IHC (>50%, >10-50%, ≤10%) are shown by the different columns.

ER-IHC	Tumour grade	10% or lower		10-50%		>50%		
		%	n	%	n	%	n	N total
**Uterine**	Grade 1-2<br>Grade 3	-<br>-	-<br>-	-<br>50,0	-<br>2	100<br>50,0	7<br>2	7<br>4
**Hematogenous**	Grade 1-2<br>Grade 3	8,3<br>-	1<br>-	8,3<br>-	1<br>-	83,3<br>100	10<br>1	12<br>1
**Lymphogenic**	Grade 1-2<br>Grade 3	14,3<br>-	1<br>-	14,3<br>25,0	1<br>1	71,4<br>75,0	5<br>3	7<br>4
**Intra-abdominal**	Grade 1-2<br>Grade 3	14,3<br>-	1<br>-	-<br>-	-<br>-	85,7<br>-	6<br>-	7<br>0
**Port-site**	Grade 1-2<br>Grade 3	-<br>-	-<br>-	-<br>-	-<br>-	100<br>-	3<br>-	3<br>0
**Vaginal vault**	Grade 1-2<br>Grade 3	-<br>-	-<br>-	8,3<br>-	2<br>-	91,7<br>100	22<br>2	24<br>2

Supplementary Table S1. B. PR-IHC in different tumour locations stratified by tumour grade. Predetermined cut-offs of PR-IHC (>50%, >10-50%, ≤10%) (*as the previously identified optimal cut-off value in the PROMOTE study* [24]) are shown by the different columns.

PR-IHC	Tumour grade	10% or lower		10-50%		>50%		
		%	n	%	n	%	n	N total
**Uterine**	Grade 1-2<br>Grade 3	-<br>25,0	-<br>1	-<br>25,0	-<br>1	100<br>50,0	7<br>2	7<br>4
**Hematogenous**	Grade 1-2<br>Grade 3	25,0<br>-	3<br>-	8,3<br>-	1<br>-	66,7<br>100	8<br>1	12<br>1
**Lymphogenic**	Grade 1-2<br>Grade 3	28,6<br>50,0	2<br>2	28,6<br>25,0	3<br>1	42,9<br>25,0	3<br>1	7<br>4
**Intra-abdominal**	Grade 1-2<br>Grade 3	14,3<br>-	1<br>-	14,3<br>-	1<br>-	71,4<br>-	5<br>-	7<br>-
**Port-site**	Grade 1-2<br>Grade 3	33,3<br>-	1<br>-	33,3<br>-	1<br>-	33,3<br>-	1<br>-	3<br>-
**Vaginal vault**	Grade 1-2<br>Grade 3	20,0<br>-	5<br>-	28,0<br>50,0	7<br>1	52,0<br>50,0	13<br>1	25<br>2

Supplementary Table S1. C. ERPAS in different tumour locations stratified by tumour grade. Predetermined cut-offs of ERPAS (>15, ≤15) (*as the previously identified optimal cut-off value in the PROMOTE study* [24]) are shown by the different columns.

ERPAS	Tumour grade	15 or lower		>15		
		%	n	%	n	N total
**Uterine**	Grade 1-2	42,9	3	57,1	4	7

	Grade 3	25,0	1	75,0	3	4
**Hematogenous**	Grade 1-2	37,5	3	62,5	5	8
	Grade 3	-	-	100	1	1
**Lymphogenic**	Grade 1-2	71,4	5	28,6	2	7
	Grade 3	66,7	2	33,3	1	3
**Intra-abdominal**	Grade 1-2	40	2	60	3	5
	Grade 3	-	-	-	-	0
**Port-site**	Grade 1-2	100	3	-	-	3
	Grade 3	-	-	-	-	0
**Vaginal vault**	Grade 1-2	44,0	11	56,0	14	25
	Grade 3	100	2	-	-	2
